# A blue-shifted anion channelrhodopsin from the Colpodellida alga *Vitrella brassicaformis*

**DOI:** 10.1038/s41598-023-34125-8

**Published:** 2023-04-28

**Authors:** Keiichi Kojima, Shiho Kawanishi, Yosuke Nishimura, Masumi Hasegawa, Shin Nakao, Yuya Nagata, Susumu Yoshizawa, Yuki Sudo

**Affiliations:** 1 Faculty of Medicine, Dentistry and Pharmaceutical Sciences, Okayama University, Okayama, 700-8530 Japan; 2 Graduate School of Medicine, Dentistry and Pharmaceutical Sciences, Okayama University, Okayama, 700-8530 Japan; 3 Research Center for Bioscience and Nanoscience (CeBN), Research Institute for Marine Resources Utilization, Japan Agency for Marine-Earth Science and Technology (JAMSTEC), Kanagawa, 237–0061 Japan; 4 Institute for Extra-Cutting-Edge Science and Technology Avant-Garde Research (X-Star), Japan Agency for Marine-Earth Science and Technology (JAMSTEC), Kanagawa, 237–0061 Japan; 5 Atmosphere and Ocean Research Institute, The University of Tokyo, Chiba, 277-8564 Japan

**Keywords:** Ion transport, Biophysical chemistry, Ion channels, Bioenergetics

## Abstract

Microbial rhodopsins, a family of photoreceptive membrane proteins containing the chromophore retinal, show a variety of light-dependent molecular functions. Channelrhodopsins work as light-gated ion channels and are widely utilized for optogenetics, which is a method for controlling neural activities by light. Since two cation channelrhodopsins were identified from the chlorophyte alga *Chlamydomonas reinhardtii*, recent advances in genomic research have revealed a wide variety of channelrhodopsins including anion channelrhodopsins (ACRs), describing their highly diversified molecular properties (e.g., spectral sensitivity, kinetics and ion selectivity). Here, we report two channelrhodopsin-like rhodopsins from the Colpodellida alga *Vitrella brassicaformis*, which are phylogenetically distinct from the known channelrhodopsins. Spectroscopic and electrophysiological analyses indicated that these rhodopsins are green- and blue-sensitive pigments (λ_max_ =  ~ 550 and ~ 440 nm) that exhibit light-dependent ion channeling activities. Detailed electrophysiological analysis revealed that one of them works as a monovalent anion (Cl^−^, Br^−^ and NO_3_^−^) channel and we named it *V. brassicaformis* anion channelrhodopsin-2, VbACR2. Importantly, the absorption maximum of VbACR2 (~ 440 nm) is blue-shifted among the known ACRs. Thus, we identified the new blue-shifted ACR, which leads to the expansion of the molecular diversity of ACRs.

## Introduction

Microbial rhodopsins are a family of photoreceptive membrane proteins that play important roles in the photoreception of microorganisms, regulating their energy production and phototactic responses^1–3^. Rhodopsins commonly contain 7 transmembrane α-helices and a derivative of vitamin-A, retinal, as a chromophore to absorb light ranging from visible to the near-infrared region (i.e., 440–690 nm)^4^. In the unphotolyzed state, all-*trans* retinal is covalently bound to a conserved Lys residue located in the seventh helix (called helix G) through a protonated Schiff base linkage. The protonated Schiff base is stabilized by negatively charged counterion(s) (Glu and/or Asp)^1,2^. In general, light absorption triggers the isomerization of the chromophore retinal from the all-*trans* to the 13-*cis* form. After photoisomerization, rhodopsins exhibit several photointermediates (e.g., K, L, M, N and O intermediates in *Halobacterium salinarum* bacteriorhodopsin, HsBR), which have distinctive photochemical features after which they return to the original unphotolyzed state with the re-isomerization of the retinal. During that cyclic series of reactions (called the photocycle), conformational changes of the protein moiety and continuous p*K*_a_ changes of the charged amino acids, including the protonated Schiff base and its counterion, occur to induce the cognate light-dependent molecular functions^1,2^. So far, more than 7000 microbial rhodopsins have been identified from archaea, bacteria, eukaryotic microorganisms and viruses that have a variety of distinct molecular functions, such as ion pumps, ion channels, phototactic sensors and enzymes, based on recent advances in genomics and bioinformatics^1–3^. The structure–function relationship among various rhodopsins has been analyzed using a variety of biophysical and biochemical methods to understand the molecular mechanisms of their functions^1–3^.

**Figure 1:**
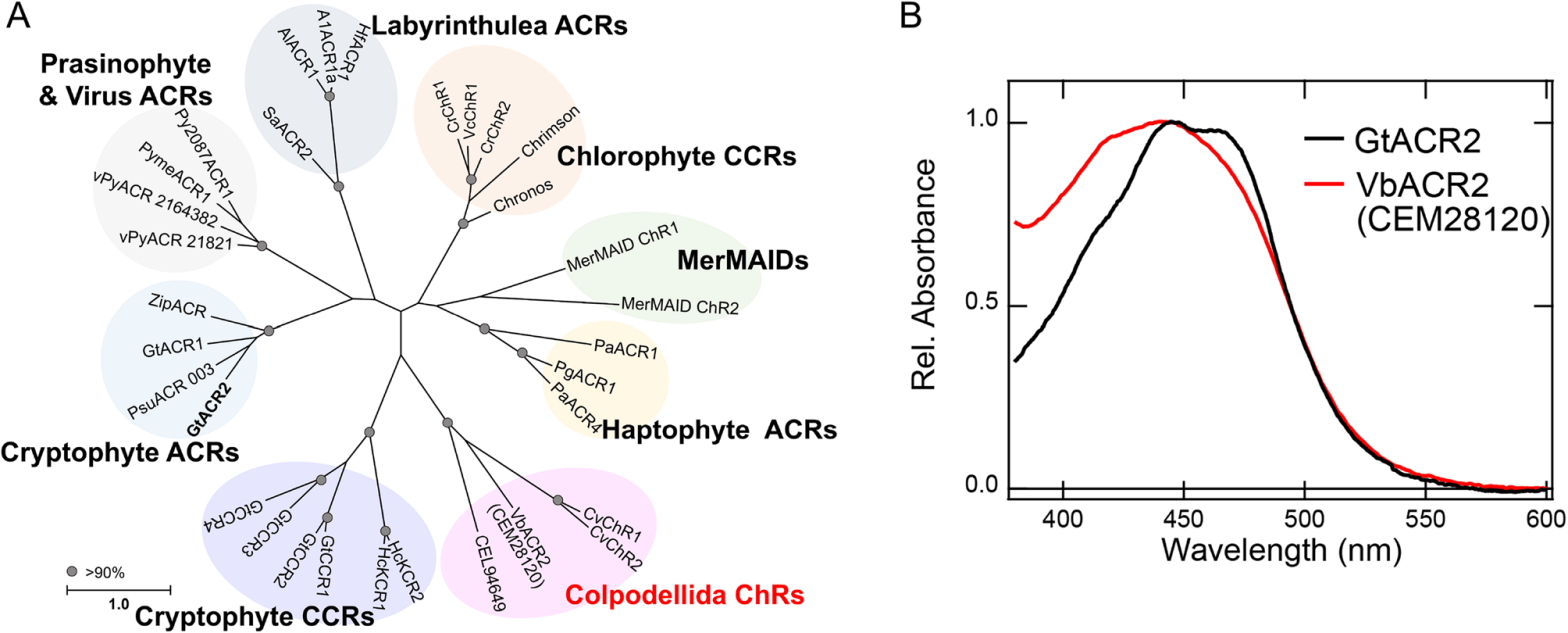
Phylogenetic relationship of channelrhodopsins and absorption spectra of VbACR2 and GtACR2. (**A**) The phylogenetic tree of known channelrhodopsins and Colpodellida ChRs [CEL94649 and VbACR2 (CEM28120)] with gray circles representing bootstrap values > 90%. The scale bar represents the average number of amino acid substitutions per site. The phylogenetic tree was inferred using the maximum likelihood method of MEGA X software (version 10.2.2)^57^. Accession numbers of the sequence data in the tree are as follows: CrChR1, AAL08946; CrChR2, AAM15777; VcChR1, AAZ66339; Chronos, AHH02106; Chrimson, AHH02126; GtCCR1, ANC73520; GtCCR2, ANC73518; GtCCR3, ANC73519; GtCCR4, ARQ20888; HcKCR1, MZ826861; HcKCR2, MZ826862; GtACR1, KP171708; GtACR2, KP171709; ZipACR, APZ76709; PsuACR_003, APZ76711; PymeACR1, MT353682; Py2087ACR1, QNU12852; vPyACR_21821, QNU12854; vPyACR_2164382, QNU12855; MerMAID ChR1, QCW06519; MerMAID ChR2, QCW06520; PgACR1, MT002472; PaACR1, MT002471; PaACR4, MT002464; AlACR1, MT002467; A1ACR1a, MT002468; HfACR1, MT002469; and SaACR2, MT002463. (**B**) The absorption spectra of the purified VbACR2 (CEM28120) in SMA lipid particles and GtACR2 in DDM micelles. The spectra were normalized at peak absorbance.

In 2002 and 2003, two light-gated cation channels, channelrhodopsin-1 and -2 (CrChR1 and CrChR2, respectively), were identified from the chlorophyte alga *Chlamydomonas reinhardtii*^5–7^. CrChR1 and CrChR2 absorb green and blue light (absorption maximum, λ_max_ =  ~ 510 and ~ 470 nm, respectively) and transport cations (e.g., H^+^ and Na^+^), resulting in phototactic responses through an intracellular signaling cascade^5–7^. Of note, they can induce membrane depolarization through their inward cation (mainly Na^+^) transport. In 2005, Boyden et al. produced rat hippocampal neurons expressing CrChR2 and successfully induced light-dependent neural activation via membrane depolarization on a msec time scale^8^. The technology to regulate biological phenomena (mainly neural activity) by light-sensitive molecules is named optogenetics^9,10^. As a powerful method to noninvasively control neural activity with a high spatiotemporal resolution, optogenetics has been progressively developed in the life sciences field. After the discovery of CrChR1 and CrChR2, a variety of natural cation channelrhodopsins (CCRs) with characteristic properties (e.g., fast and slow channel closing, blue- and red-shifted spectral sensitivities and large photocurrents) has been discovered from chlorophyte and cryptophyte algae^9,11–16^ (Fig. 1A).

In contrast to CCRs, two natural anion channelrhodopsins (ACRs) were identified in 2015 from the cryptophyte alga *Guillardia theta* and named GtACR1 and GtACR2 (Fig. 1A)^17^. GtACR1 and GtACR2 absorb green and blue light (λ_max_ =  ~ 510 and ~ 470 nm) respectively, and selectively transport monovalent anions (e.g., Cl^−^, Br^−^ and NO_3_^−^), which can induce membrane hyperpolarization through their inward anion (mainly Cl^−^) transport in neurons. Thus, GtACR1 and GtACR2 are widely utilized as tools for high-performance neural silencers^18^. Since then, a variety of natural ACRs with characteristic properties have been discovered not only from algae (e.g., cryptophyte, haptophyte, prasinophyte and dinoflagellate algae) but also from heterotrophic protists labyrinthulea and marine giant viruses (Fig. 1A)^12,19–23^. For instance, ZipACR (also called PsuACR_973) from *Proteomonas sulcate* shows fast channel closing kinetics (~ 2.2 ms) and has been used for high frequency neural silencing^20^. A family of ACRs from labyrinthulea (named RubyACRs) shows red-shifted λ_max_ (590–610 nm) and can be used for neural silencing by red light^22^. Furthermore, a family of ACRs metagenomically identified from marine microorganisms (named MerMAIDs) shows a rapid and large desensitization of photocurrents during continuous light illumination and they have been used as neural silencers for the transient suppression of individual action potentials^21^. Thus, identifying new channelrhodopsins from nature is a powerful strategy to expand their molecular diversity in addition to the production of genetically modified variants of CCRs and ACRs^9,10,12^.

Based on that background, we focused on putative microbial rhodopsins from Colpodellida in this study. Colpodellida is a photosynthetic algae that is closely related to the protozoan phylum Apicomplexa and lives in association with corals^24,25^. Since the protozoan phylum Apicomplexa evolved from photosynthetic ancestors, Colpodellida is believed to be a good example of a bridge linking algae (botanical protists) and protozoa (zoological protists). In 2015, the nuclear genomes of two Colpodellida species, *Vitrella brassicaformis* and *Chromera velia*, were sequenced^25^, in which channelrhodopsin-like genes were identified^12,26^. In this study, we refer to these putative rhodopsins as “Colpodellida ChRs”, which form a distinct family from the known CCRs and ACRs in the phylogenetic tree (Figs. 1 and S1). We have now expressed two rhodopsins from *V. brassicaformis* (Genbank accession numbers: CEL94649 and CEM28120) as recombinant proteins in mammalian cells in culture and performed spectroscopic and electrophysiological analyses. The results indicate that they are green- and blue-sensitive pigments (λ_max_ = ~ 550 and ~ 440 nm), respectively, and both show light-dependent ion channeling activity. More detailed analysis revealed that one of them (CEM28120) works as a blue-sensitive monovalent anion (Cl^−^, Br^−^ and NO_3_^−^) channel and we named it *V. brassicaformis* anion channelrhodopsin-2 (VbACR2). Of note, the λ_max_ of VbACR2 (~ 440 nm) is blue-shifted among the known ACRs (λ_max_ = 445–610 nm). Based on these results, we compared the molecular properties of VbACR2 with a typical blue-shifted ACR, GtACR2 and discuss the optogenetic potential of VbACR2.

## Results and discussion

### Absorption spectra of *V. brassicaformis* channelrhodopsin-like proteins

To investigate the cDNAs of two channelrhodopsin-like genes from *V. brassicaformis* (CEL94649 and CEM28120) that encode photoactive proteins, we expressed them as recombinant proteins in mammalian HEK293 cells, which have been used for the functional expression of animal rhodopsins and eukaryotic microbial rhodopsins^27–30^. As shown below, since the recombinant CEM28120 protein shows a blue-sensitive anion channeling activity like GtACR2, we refer to the channelrhodopsin-like protein (CEM28120) as *V. brassicaformis* anion channelrhodopsin-2 (VbACR2).

For the sample preparation, we solubilized HEK293 cells expressing those genes using the detergent n-dodecyl-*β*-D-maltoside (DDM) and purified the recombinant proteins using a Ni^2+^ affinity column chromatography system that has been widely used for the purification of various microbial rhodopsins including CCRs and ACRs^19,30,31^. However, we didn’t obtain visible light-sensitive pigments using that method. Thus, we used styrene-maleic acid (SMA) copolymers as an alternative to DDM since they spontaneously form nanoscale lipid particles containing membrane proteins and associated lipids in the absence of detergent, and can be used to characterize membrane proteins^32–34^. In fact, SMA has been used to characterize several kinds of microbial rhodopsins including an unstable *Halobacterium salinarum* sensory rhodopsin I, and it has been reported that the photochemical properties of rhodopsins are similar to those observed in a membrane environment^35^. Thus, we solubilized HEK293 cells using 5% (w/v) SMA and purified the extract using a Ni^2+^ affinity column chromatography system according to a previously reported method^35,36^. Figures S2A and 1B show the absorption spectra of the purified recombinant proteins CEL94649 and VbACR2 (CEM28120) in SMA lipid particles. The peak wavelengths of the absorption spectra of CEL94649 and VbACR2 were 550 and 440 nm, respectively. Those results indicate that these cDNAs encode green- and blue-sensitive photoactive proteins. Of note, the λ_max_ of VbACR2 (i.e., 440 nm) is blue-shifted as compared with that of GtACR2 (λ_max_ =  ~ 460 nm) (Fig. 1B)^30^, which suggests that VbACR2 is categorized as a blue-shifted channelrhodopsin. It is noted that the spectrum of VbACR2 is broader than that of GtACR2 (Fig. 1B). Since we directly solubilized HEK293 cells using SMA, it is suggested that SMA forms the nanoscale lipid particles containing not only target proteins (i.e., VbACR2) but also other endogenous proteins expressing in HEK293 cells (e.g., hemoprotein). We speculate that the broader spectrum of VbACR2 is derived from the contaminated other proteins (i.e., low purity of the sample).

### Ion transport activity of *V. brassicaformis* channelrhodopsin-like proteins

**Figure 2:**
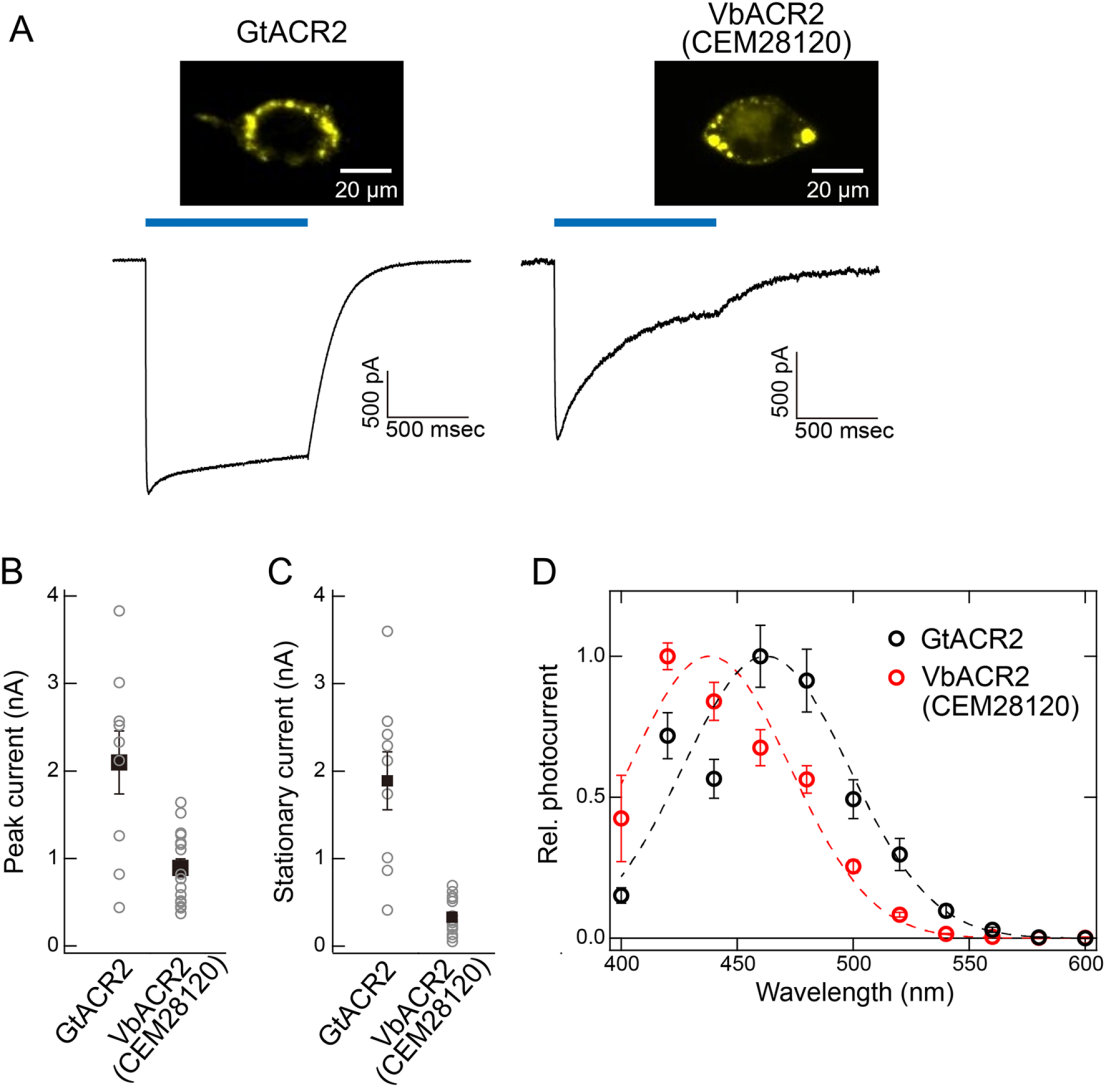
Expression and electrophysiological measurements of VbACR2 and GtACR2. (**A**) Representative photocurrent traces of GtACR2 and VbACR2 (CEM28120) elicited with blue light (blue bars) at the membrane potential of − 60 mV in the standard intracellular and extracellular solutions (146 and 147 mM Cl^−^ in the intracellular and extracellular solution, respectively) (Table S1). Fluorescence images of ND7/23 cells expressing GtACR2 and VbACR2 were also shown. The fluorescence signals derived from EYFP which was fused to the C-terminus of GtACR2 and VbACR2 were monitored. (**B**, **C**) Peak (**B**) and stationary current amplitudes (**C**) of GtACR2 and VbACR2 elicited with blue light (470–495 nm, 2.0 mW mm^−2^) at the membrane potential of − 60 mV in the standard solutions. The data are mean values ± SEM (n = 9–16 cells). (**D**) Photocurrent action spectra of GtACR2 and VbACR2. The peak current amplitudes were plotted against the wavelength of light. The data are mean values ± SEM (n = 5 cells).

To determine the molecular functions of the two channelrhodopsin-like proteins, CEL94649 and VbACR2 (CEM28120), we performed electrophysiological analysis that is conventional method used for ion transport rhodopsins^6,7,17,30^. We expressed those rhodopsins in mammalian ND7/23 cells, in which the cDNAs of CEL94649 and VbACR2 were inserted downstream of the CMV promoter with a trafficking signal (TS) and an endoplasmic reticulum export signal (ER) to enhance the membrane localization as previously described^29,37^. In the transfected cells, yellow fluorescence signals from EYFP were observed around the plasma membrane, which indicates the expression of the cognate rhodopsin in the plasma membrane (Figs. S2B and 2A). However, several bright spots of fluorescence were also observed around the intracellular region, which suggests that several molecules were not transported to the plasma membrane and formed aggregates in the intracellular region. We then measured the photocurrents of CEL94649 and VbACR2 illuminated with green and blue light, respectively (Figs. S2B and 2A). As a control, we also measured the photocurrents of GtACR2, which is a typical blue-shifted ACR, using the same expression system (Fig. 2A). With our standard solutions for electrophysiological recording (146 and 147 mM Cl^−^ in the intracellular and extracellular solution, Tables S1 and S2), inward photocurrents were generated by CEL94649 and VbACR2 at the holding potential of − 60 mV (Figs. S2B and 2A). These data clearly indicate that these rhodopsins transport ions in a light-dependent manner. The average peak current of VbACR2 (0.89 nA) was 2.4-fold lower than that of GtACR2 (2.1 nA) while that of CEL94649 (59 pA) was much smaller (Figs. 2B and S2C). It is known that the photocurrents of most known ChRs decline from an initial peak current to a lower stationary level. This process is called desensitization and observed in GtACR2 and VbACR2 (Fig. 2A). We also compared the stationary currents of VbACR2 and GtACR2 (Fig. 2C). The average stationary current of VbACR2 (0.33 nA) was 5.7-fold lower than that of GtACR2 (1.9 nA). Thus, GtACR2 showed higher peak and stationary photocurrents as compared with VbACR2 in ND7/23 cells. Since the λ_max_ of absorption spectrum of VbACR2 was estimated to be 440 nm (Fig. 1B), the photocurrent action spectrum of VbACR2 was recorded. We measured photocurrents of VbACR2 and GtACR2 illuminated with different wavelengths of light between 400 and 600 nm (20 nm intervals) at the holding potential of − 60 mV, and plotted the peak photocurrents against the wavelength of light (Figs. 2D and S3). In Fig. S3, the peak current amplitudes were plotted together with the absorption spectra of VbACR2 and GtACR2. As seen, the absorption spectra roughly matched well with the plots of the amplitudes in both rhodopsins. Then, the data of the peak current amplitudes were fitted by a normal Gaussian distribution to estimate the λ_max_ of the action spectra (Fig. 2D). The λ_max_ of action spectrum of GtACR2 was estimated to be 460 nm, which is consistent with the previous electrophysiological study^17^, while that of VbACR2 was estimated to be 440 nm. Thus, the photocurrent action spectrum of VbACR2 was blue-shifted as compared with GtACR2, which clearly implies that VbACR2 is categorized into blue-shifted channelrhodopsins, such as TsChR (λ_max_ = 440 nm), C1ACR_023 (λ_max_ = 445 nm) and GtACR2 (λ_max_ = 470 nm)^13,17,20^. It should be noted that the Gaussian curves were not employed for the fitting of the action spectra of channelrhodopsin (including GtACR2), probably due to their asymmetry. In fact, the points at 440 nm are low deviating from the Gaussian curves (Fig. 2D). To more accurately estimate the shapes and λ_max_ of action spectra, it is necessary to measure the photocurrents illuminated with different wavelengths of light between 400 and 600 nm at short intervals.

**Figure 3:**
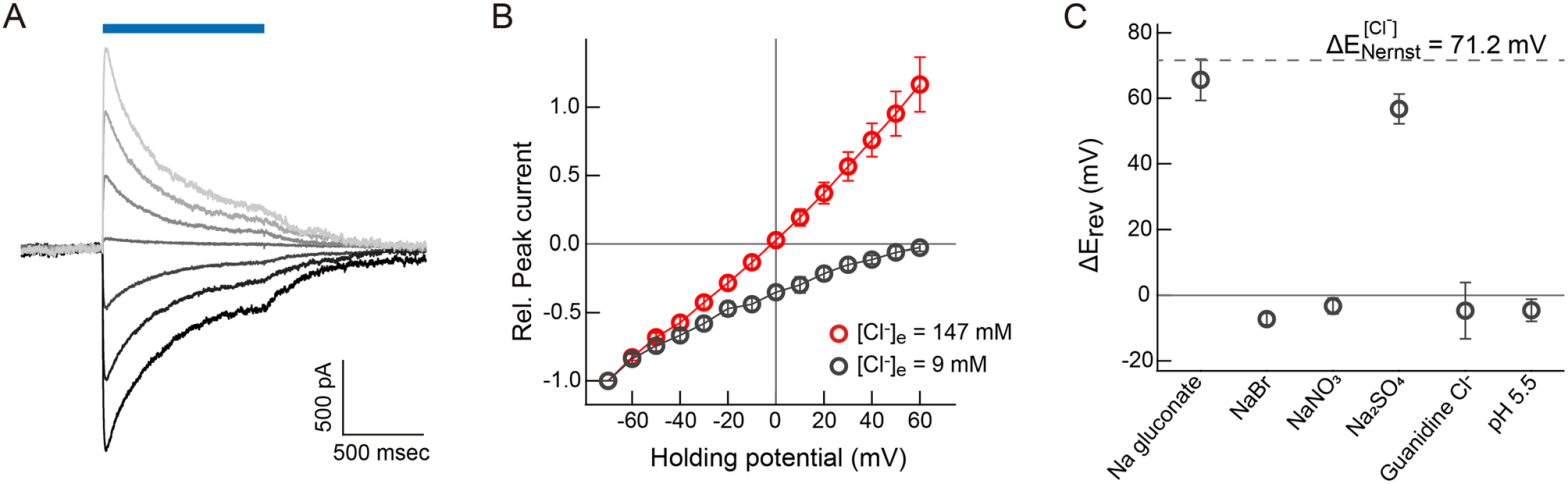
Ion selectivity of VbACR2. (**A**) Representative photocurrent traces of VbACR2 (CEM28120) elicited with blue light (blue bar) at different membrane potentials (− 60 to + 60 mV, in 20 mV steps, from the bottom to the top) in standard solutions (146 and 147 mM Cl^−^ in the intracellular and extracellular solutions, respectively). (**B**) Current–voltage relationship of the VbACR2 peak photocurrent at 147 mM (red) and 9 mM (gray) extracellular chloride ([Cl^−^]_e_). The chloride concentration was reduced by replacing NaCl with sodium gluconate in the extracellular solution (Table S2). Peak currents were normalized at the membrane potential of − 70 mV. The data are mean values ± SEM (n = 7 cells). (**C**) ΔE_rev_ values of VbACR2 upon exchange of the extracellular solution. The data are mean values ± SEM (n = 5–7 cells).

We then measured the photocurrents at different membrane potentials (-70 to + 60 mV, in 10 mV steps) to determine whether they work as ion channels or ion pumps (Figs. 3A and S2D). The directions of photocurrents of both CEL94649 and VbACR2 were reversed at the holding potential of around 0 mV, which clearly indicates that both work as ion channels (Figs. 3A and S2D). We then estimated the reversal potential and ion selectivity of CEL94649 and VbACR2. As seen in Figs. 3B and S2E, the reversal potential (E_rev_: the potential where the net ion flux is zero) was almost zero at 146 and 147 mM intracellular and extracellular Cl^−^, respectively, which suggests that CEL94649 and VbACR2 transport Cl^−^. Since the amplitudes of photocurrent of CEL94649 were very small (Fig. S2C), probably due to the low membrane localization, we examined the ion selectivity of VbACR2 but not CEL94649. We then measured the photocurrents of VbACR2 from − 70 to + 60 mV where the extracellular Cl^−^ concentration ([Cl^−^]_e_) was reduced to 9 mM by replacing NaCl with sodium gluconate while maintaining the intracellular Cl^−^ concentration ([Cl^−^]_i_) at 146 mM (Fig. 3B,C). That replacement increased the inward current and induced a positive shift of the E_rev_. The shift of the E_rev_ (ΔE_rev_) was estimated as 65.6 mV, which is close to the theoretical Cl^–^ Nernst potential (71.2 mV) (Fig. 3B,C). Thus, these data clearly indicate that VbACR2 works as a light-dependent anion (Cl^−^) channel. As we used the standard intracellular solution containing 10 mM Na^+^ which mimics the intracellular low Na^+^ concentration (e.g., 10–15 mM) in mammalian cells, it should be noted that a large inward Na^+^ gradient was present when we used the standard extracellular solution containing 138 mM Na^+^ (Tables S1 and S2). We also investigated the current–voltage relationship of VbACR2 with the Na^+^ intracellular solution containing 150 mM Na^+^ (Table S1). The replacement of NaCl with sodium gluconate in the extracellular solution increased the inward current and induced a positive shift of the E_rev_ (Fig. S4). ΔE_rev_ was estimated as 67.2 mV when we used the Na^+^ intracellular solution (Table S1), which is also close to the theoretical Cl^–^ Nernst potential (71.2 mV). This data suggests that the low Na^+^ concentration in the standard intracellular solution (Table S1) did not cause a significant effect on the ΔE_rev_. Since the CEM28120 protein shows a blue-sensitive anion channeling activity like GtACR2, we refer to CEM28120 as *V. brassicaformis* anion channelrhodopsin-2, VbACR2. On the other hand, the photocurrent of the CEL94649 protein was too small to quantitatively investigate whether it works as a cation or anion channel. The residue at the position of Asp85 in HsBR, the Schiff base proton acceptor in HsBR, is a neutral residue (mainly Ser) in all the ACRs so far identified^26^. VbACR2 has an uncharged residue (Gln128) at this position similar to the known ACRs, but the Gln residue seems to be unique (Fig. S1). On the other hand, CEL94649 has Glu96 at this position as do nearly all CCRs (with few exceptions: Met140 of Chronos and Ala178 of DsChR), but no ACRs (Fig. S1)^9,13,26^. This suggests that CEL94649 works a cation channel. As a future work, further electrophysiological experiments will be required to determine the substrates of CEL94649 by using its truncated mutants and different expression system (e.g., modification of gene construction and changing cell lines) to increase the photocurrent signals.

**Figure 4:**
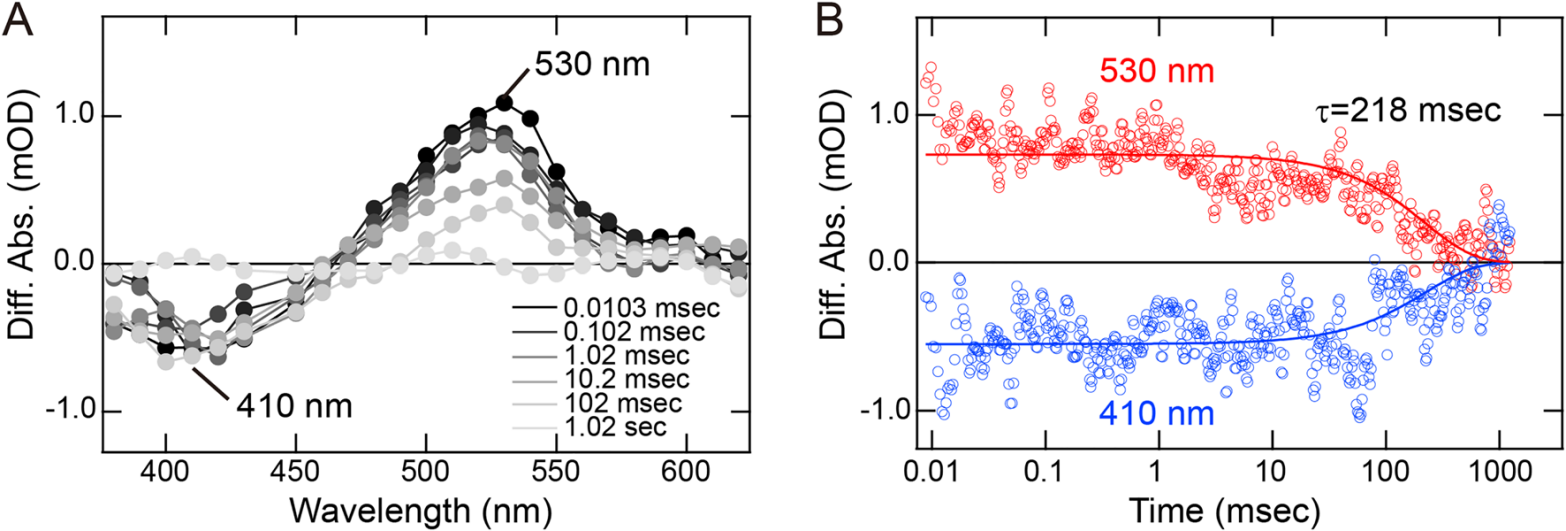
Photoreaction kinetics of VbACR2. (**A**) Flash-induced difference absorption spectra of VbACR2 (CEM28120) over a time range of 0.0103 ms to 1.02 s. Depletion of the original state (410 nm) and simultaneous increases in the photointermediate P_530_ (530 nm) were observed. (**B**) Flash-induced kinetic data at 410 and 530 nm representing the recovery of the original state and the decay of P_530_, respectively. The plots were well-fitted to a single exponential equation, and the time constant was estimated to be 218 ms.

To evaluate the conductance of other anions, we analyzed the current–voltage relationship of VbACR2 by replacing Cl^−^ with Br^−^, NO_3_^−^ and SO_4_^2−^ in the extracellular solution (Figs. 3C and S5). The replacement with Br^−^ and NO_3_^−^ resulted in small ΔE_rev_ shifts (− 7.2 and − 3.3 mV, respectively) (Fig. 4C), indicating that VbACR2 transports Br^−^ and NO_3_^−^ as well as Cl^−^, as previously reported for other ACR^17,21,23^. On the other hand, the replacement with SO_4_^2−^ resulted in a large positive ΔE_rev_ shift (56.7 mV) as did the replacement of NaCl with sodium gluconate (Fig. 3C), indicating that VbACR2 doesn’t transport the divalent anion SO_4_^2−^, as previously reported for other ACRs^17^. These results revealed the nonselective conductivity of monovalent anions with a similar permeability (Cl^−^ ≈ Br^−^ ≈ NO_3_^−^). So far, it has been reported that the known ACRs, such as GtACR1 and GtACR2, show higher permeabilities of Br^−^ and NO_3_^−^ compared with Cl^−^^17,21^. Therefore, a high Cl^−^ selectivity is a characteristic feature for VbACR2 among ACRs. Then, Na^+^ ions were substituted for guanidine and the extracellular pH was lowered by changing the extracellular solution (i.e., increases in H^+^ concentration) to investigate the effects of cations (Na^+^ and H^+^) on the current–voltage relationship of VbACR2 (Figs. 3C and S5). The substitution and decrease of pH resulted in small ΔE_rev_ shifts (− 4.7 and − 4.5 mV, respectively) (Fig. 3C), which excludes the possibility that the cations are transported by VbACR2. Therefore, we concluded that VbACR2 works as a monovalent anion channel with a high anion selectivity as well as other ACRs^17,21,23^.

### Photocycle of VbACR2

Channelrhodopsins show ion channeling functions by opening and then closing the ion-permeable channel during the photocycle process after light absorption. To investigate the photocycle of VbACR2, we performed time-resolved flash photolysis analyses using the purified sample in SMA lipid particles. Figure 4A shows the flash-induced difference spectra over the spectral range of 380–620 nm and the temporal range from 10.3 μs to 1.02 s. Within 10 µs (0.01 ms) after photoexcitation, a negative peak and a concomitant positive peak were observed at around 410 nm and 530 nm (Fig. 4A,B), which reflects the depression of the original unphotolyzed state and the formation of a red-shifted photointermediate (named P_530_), respectively. The decay of P_530_ and the concomitant recovery of the original state were observed within ~ 1 s (Fig. 4A,B). Figure 4B shows the time-dependent absorbance changes at 410 and 530 nm for monitoring the original state and P_530_, respectively. The decay rate of P_530_, that is the recovery rate of the original state, was estimated as 218 ms by fitting with a single exponential function (Fig. 4B). On the other hand, we did not detect the formation of other photointermediates such as a blue-shifted M intermediate. The formation of M intermediate, in which the Schiff base has transferred its proton to an acceptor in the protein, is observed in all wild-type ACRs so far studied^21,30,38^. The previous structural and spectroscopic studies proposed that the Schiff base is required to be protonated to form the Cl^−^ conductive channel, and deprotonation of the Schiff base (i.e., M intermediate formation) is coupled to closing the channel^38–41^. This model is supported by the S97E mutant of GtACR1 that has a proton acceptor placed in the position of the Glu or Asp acceptor in CCRs^38^. In fact, the mutant undergoes rapid proton transfer (i.e., M intermediate formation) and the channel does not open. Difference absorption spectra of VbACR2 did not show the apparent positive peak around 400 nm (i.e., M intermediate formation) in Fig. 4A. However, there is a possibility that the absorbance changes derived from M intermediate formation is canceled by the depression of the initial state of VbACR2 in the UV–visible spectroscopic analysis since the λ_max_ of M intermediate and initial state are similar (~ 400 and 440 nm, respectively). Therefore, further experiments (e.g., vibrational spectroscopy) is needed to detect the deprotonation of the Schiff base during the M intermediate formation process.

### Molecular configuration of VbACR2 around the retinal chromophore

In microbial rhodopsins, the protonated Schiff base is stabilized by a counterion, which enables it to absorb visible light^1^. Thus, we checked the amino acid sequences of CEL94649 and VbACR2 (CEM28120) at positions 99, 128 and 257 in VbACR2, which are the residues corresponding to Glu68 (the counterion in GtACR1), Asp85 and Asp212 (primary and secondary counterions, respectively, in HsBR), as counterion candidates for CEL94649 and VbACR2^1,39^. Among those three positions, Glu96 and Asp257 are the only carboxylate residues in CEL94649 and VbACR2, respectively (Fig. S1). This suggests that Glu96 of CEL94649 and Asp257 of VbACR2 work as counterions. In addition, there is a possibility that a chloride ion is bound in the dark state and function as a counter ion in CEL94649 and VbACR2. It is needed to remove chloride ions from the purified sample of CEL94649 and VbACR2 and perform the spectroscopic analysis for investigation of the roles of chloride ion.

**Figure 5:**
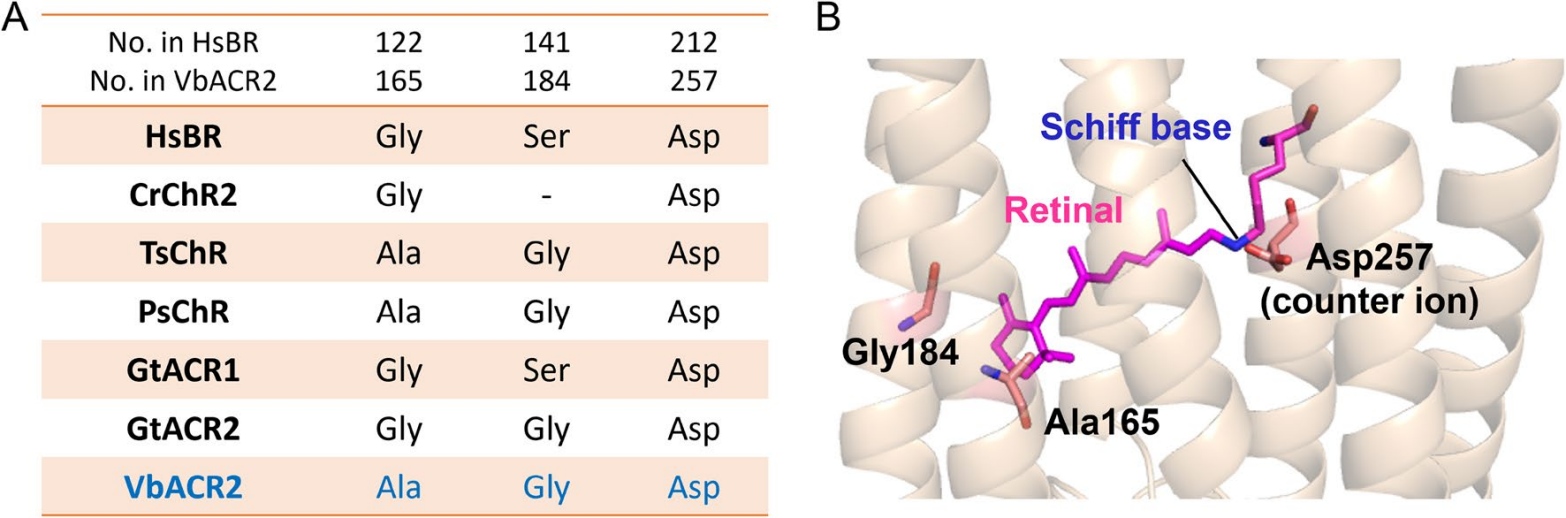
Hypothetical mechanism of the blue-shifted property of VbACR2. (**A**) Comparison of amino acid residues around the retinal. (**B**) AlphaFold structural model for VbACR2 (https://alphafold.ebi.ac.uk/). The putative key residues of VbACR2 for its color tuning (Ala165 and Gly184) are indicated on the model structure (orange sticks). The putative counterion (Asp257) is indicated (orange stick). The retinal and the Schiff base linkage with Lys residue of the crystal structure of GtACR1 are shown (magenta sticks).

Since 2015, various kinds of natural ACRs and their genetically modified variants have been identified and produced^17,20–23,26,30,42,43^. Among them, the λ_max_ values are located from 445 (C1ACR_023) to 610 nm (A1ACR1 and HfACR1)^17,19–23^. Thus, VbACR2 (λ_max_ =  ~ 440 nm) can be categorized as a blue-shifted ACR. The λ_max_ of microbial rhodopsins is explained by the energy gap between the ground and excited states of retinal in the protein moiety^44,45^. The energy gap is attributed to several factors, such as the planarity of the polyene chain and the charge distribution of π electrons of the retinal, which is mainly regulated by amino acid residues around the retinal. For instance, it is known that the planarity/torsion at the C_6_–C_7_ bond, which connects the β-ionone ring with the polyene chain of the retinal, is an important factor that determines the λ_max_ both in microbial and in animal rhodopsins^46,47^. In fact, we previously produced blue-shifted variants of C1C2 (a chimeric protein between CrChR1 and CrChR2) and a proton pump rhodopsin from *Halorubrum sodomense* (Archaerhodopsin-3, AR3) by introducing amino acid substitutions (i.e., G122A and S141G in the HsBR numbering system) that enhance the torsion around the C_6_–C_7_ bond of the retinal^47^. In addition, the replacement of Gly152 in GtACR2 (Ser141 in HsBR) with a Ser residue induced a spectral red-shift, which indicates that Gly152 plays an important role in maintaining the blue-light sensitivity of GtACR2 (λ_max_ = 460 nm)^30^. Furthermore, quantum mechanical/molecular mechanical calculations predicted that the replacement of Ser156 in GtACR1 (Ser141 in HsBR) with Gly stabilizes the S_0_ (ground) state and induces its spectral blue-shift, which indicates that Ser156 plays an important role in maintaining the green-light sensitivity of GtACR1 (λ_max_ = 515 nm)^48^. Comparison of the amino acid sequences and structures between VbACR2 and other typical rhodopsins indicated that Ala165 and Gly184 of VbACR2 are the corresponding residues of Gly122 and Ser141 of HsBR and they are located near the β-ionone ring of the retinal (Fig. 5). The blue-shifted CCRs, such as TsChR (λ_max_ = 440 nm) and PsChR (λ_max_ = 437 nm), naturally contain Ala165 and Gly184 of VbACR2 at their corresponding positions^13,49^. Thus, VbACR2 contains Ala165 and Gly184 near the β-ionone ring of the retinal, which probably contribute to the torsion at the C_6_–C_7_ bond of the retinal for its spectral blue-shift. In addition to the effects of residues near the β-ionone ring of the retinal, it is known that the alteration in the strength of the electrostatic interaction between the protonated Schiff base and counterion affects the spectral tuning of microbial rhodopsins^50^. As mentioned above, VbACR2 has the unique Glu residue (Gln128) near the Schiff base unlike the known ACRs (Fig. S1), which could affect the electrostatic interaction between the protonated Schiff base and counterion. As a future work, we will perform mutational analysis on VbACR2 to investigate the roles of the above amino acid residues on the spectral blue-shift.

So far, a variety of color variants of CCRs and ACRs have been identified from nature and produced by mutations, which enable the optogenetic manipulation of neural activities with multi-color light^9,11,12^. Particularly, blue- and red-shifted channelrhodopsins show a small spectral overlap, which means that two-color excitation (i.e., blue and red illumination) can independently and precisely activate only blue- and red-shifted channelrhodopsins, respectively. This combination is used for independent optical activation and inhibition of two distinct neuronal populations^13^. Furthermore, various color variants of fluorescent molecules (e.g., Ca^2+^-sensors and voltage indicators) have also been used to achieve simultaneous optical perturbation and imaging in neurons (so-called all-optical electrophysiology)^51,52^. Considering that VbACR2 is a blue-shifted molecule among the natural and genetically modified ACRs, we will investigate the photocurrents of VbACR2 expressed in neurons and its neural silencing effects for its application as a blue-shifted optogenetics tool.

## Conclusion

The results of this study reveal that a microbial rhodopsin obtained from *V. brassicaformis* works as a blue-sensitive monovalent anion (Cl^−^, Br^−^ and NO_3_^−^) channel. The λ_max_ of the rhodopsin named VbACR2 (~ 440 nm) is the blue-shifted molecule among the known ACRs. Thus, we identified the new blue-shifted ACR, which leads to the expansion of the molecular diversity of ACRs.

## Methods

### Gene preparation and protein expression in HEK293 cells

cDNAs encoding the 7 transmembrane domains of CEL94649, VbACR2 and GtACR2 (Genbank accession numbers: CEL94649, CEM28120 and KP171709, amino acid residues from the 1st to the 291st, 319th and 291st position, respectively) were optimized for human codon usage and were fused to a C-terminal sequence encoding a hexahistidine-tag. The fusion products were inserted into a mammalian expression vector, pCAGGS, as previously described^29,30,53^. HEK293T cells were cultured in Dulbecco’s Modified Eagle Medium Nutrient Mixture F-12 (DMEM/F12, Gibco, Thermo Fisher Scientific, USA), supplemented with 10% fetal bovine serum, 0.0625% (w/v) penicillin and 0.01% (w/v) streptomycin under a humidified atmosphere containing 5% CO_2_ at 37 °C. The expression plasmids were transiently transfected using the calcium phosphate method^27,28^. After 1 day incubation, all-*trans*-retinal (final conc. 5 µM) was added to the transfected cells to produce the holoprotein. After another day of incubation, the cells were collected by centrifugation (6500×*g* for 10 min) at 4 °C and were resuspended in Buffer A (50 mM HEPES (pH 7.0) and 140 mM NaCl). The cells expressing CEL94649 and VbACR2 were then resuspended in Buffer B (50 mM HEPES (pH 7.4), 140 mM NaCl and 10% (w/v) glycerol), disrupted by sonication in ice-cold water and solubilized in Buffer-B containing 5% (w/v) styrene-maleic acid (SMA) copolymer for 2 h as previously described^35,36^. The solubilized fraction was collected by ultracentrifugation (115,700×*g* for 20 min) at 4 °C and the supernatant was applied to a Ni^2+^ affinity column to purify the pigments. After the column was washed with Buffer C (50 mM HEPES (pH 7.4), 140 mM NaCl, 10% (w/v) glycerol and 20 mM imidazole), the pigment was eluted with a linear gradient of imidazole using Buffer D (50 mM HEPES (pH 7.4), 140 mM NaCl, 10% (w/v) glycerol and 1 M imidazole). Purified CEL94649 and VbACR2 samples were concentrated and the buffer was exchanged using an Amicon Ultra filter to Buffer B for spectroscopic analysis. GtACR2 was purified using the detergent n-dodecyl-*β*-D-maltoside (DDM) as previously described^30^. Purified GtACR2 samples were concentrated and the buffer was exchanged using an Amicon Ultra filter to Buffer E (20 mM HEPES (pH 7.4), 300 mM NaCl, 5% (w/v) glycerol and 0.02% (w/v) DDM) for spectroscopic analysis.

### Gene preparation and protein expression in ND7/23 cells

cDNAs for CEL94649, VbACR2 and GtACR2 (amino acid residues from the 1st to the 291st, 319th and291st positions, respectively) having optimized codons for human cells were inserted into the CMV promoter-based mammalian expression vector as previously described^29,37^. Briefly, enhanced yellow fluorescent protein (EYFP) was fused to the C-terminus of rhodopsin as a reporter. In addition, EYFP was flanked with a membrane trafficking signal (TS) at the N-terminus and an endoplasmic reticulum export signal (ER) at the C-terminus to improve its expression and plasma membrane localization. The TS and ER signals were “KSRITSEGEYIPLDQIDINV” and “FCYENEV”, respectively, derived from the Kir2.1 potassium channel^54^. Furthermore, the WPRE (Woodchuck hepatitis virus Post-transcriptional Regulatory Element) sequence was inserted to stabilize the transcribed mRNA and increase the amount of translated protein^54^.

ND7/23 cells were cultured in Dulbecco’s Modified Eagle Medium Nutrient Mixture F-12 (DMEM/F12, Gibco, Thermo Fisher Scientific, USA), supplemented with 10% fetal bovine serum, 0.0625% (w/v) penicillin and 0.01% (w/v) streptomycin under a humidified atmosphere containing 5% CO_2_ at 37 °C. For rhodopsin expression, the cells were cultured on poly-lysine-coated glass (Matsunami Glass, Japan). The expression plasmids were transiently transfected using the calcium phosphate method^29,37^. After 5–6 h of incubation, all-*trans* retinal (final concentration, 1 µM) was added into the medium to produce the holoprotein. Electrophysiological analyses were conducted 48–60 h after transfection. Transfected cells were identified by the presence of EYFP fluorescence. The fluorescence signals for EYFP were observed using an IX71 inverted microscope (Olympus, Japan) with a fluorescence mirror unit (U-MYFPHQ, Olympus) and a mercury lamp (U-LH100HGAPO, Olympus). Photocurrents were measured using an EPC 10 USB computer-controlled Patch Clamp Amplifier (HEKA Elektronik, Germany) under a whole-cell patch clamp configuration^29,30^. The data were analyzed with Patch master software (HEKA Elektronik, Germany). The standard internal pipette solution for whole-cell voltage clamp recordings from ND7/23 cells contained 50 mM HEPES, 140 mM CsCl, 3 mM MgCl_2_, 5 mM Na_2_EGTA and 2.5 mM MgATP, adjusted to pH 7.3 with CsOH. To investigate the effects of intracellular Na^+^ concentrations on the ΔE_rev_, the Na^+^ internal pipette solution (50 mM HEPES, 140 mM NaCl, 3 mM MgCl_2_, 5 mM Na_2_EGTA and 2.5 mM MgATP, adjusted to pH 7.3 with CsOH) was also used (Table S1). The cells were continuously superfused by an extracellular medium (10 mM HEPES, 138 mM NaCl, 3 mM KCl, 1 mM MgCl_2_, 2 mM CaCl_2_, 0.1 M glucose, adjusted to pH 7.3 and 5.5 with NaOH). To investigate the effects of extracellular ion concentrations on ion transport activity, NaCl was also substituted by 138 mM sodium gluconate, 138 mM guanidine Cl^−^, 138 mM NaNO_3_, 138 mM NaBr and 92 mM Na_2_SO_4_ (Table S2). The detailed composition of intracellular and extracellular solutions was listed in Tables S1 and S2. Different cell culture dishes were used for electrophysiological experiments in different extracellular solution conditions. For each experimental condition, the data series were obtained using 5–16 cells. For the estimation of current–voltage relationship, the photocurrents were sequentially measured at the holding potential of − 70, − 60, − 50, − 40, − 30, − 20, − 10, 0, 10, 20, 30, 40, 50, 60 mV. The repeatability of the responses was checked by the additional measurement using the same cell. The holding voltages were corrected for liquid junction potentials (LJPs) before the recordings. LJPs were calculated using the software Liquid Junction Potential Calculator (https://swharden.com/LJPcalc/). Reversal potentials were determined based on linear fitting of the two data points crossing 0 pA or linear extrapolation from 0 pA most adjacent two data points. The cells were illuminated with a white LED (THORLABS, USA) through a band-pass filter for blue and green light illumination (470–495 and 530–550 nm, mirror unit U-FBNA and U-FGW Olympus, Japan), where the light intensities were adjusted to 2.0 and 4.4 mW mm^−2^, respectively.

For the measurement of photocurrent action spectra of VbACR2 and GtACR2, the cells were illuminated with different colors of light generated by letting the white LED (THORLABS, USA) passing through band-pass filters (400 ± 10 nm, 420 ± 10 nm, 440 ± 10 nm, 460 ± 10 nm, 480 ± 10 nm, 500 ± 10 nm, 520 ± 10 nm, 540 ± 10 nm, 560 ± 12 nm, 580 ± 10 nm, 600 ± 10 nm, THORLABS, USA) for 1 s. The light intensities were measured using an optical power meter with an optical sensor (#3664 and #9742, Hioki, Japan) and were 0.011, 0.061, 0.086, 0.047, 0.051, 0.086, 0.11, 0.21, 0.15, 0.20, 0.15 mW mm^−2^ for 400, 420, 440, 460, 480, 500, 520, 540, 560, 580, 600 nm, respectively. These values are lower than the intensities which were used for the measurement of action spectra of ChRs (e.g., 0.23 and 3.73 mW mm^−2^)^21,55^, suggesting that the values are in the linear range of the photocurrent sensitivity curve. The photocurrents were recorded at the holding potential of − 60 mV in the standard intracellular and extracellular solutions (Tables S1 and S2). The peak currents were corrected by the light intensity and then plotted against the wavelengths of light.

### Spectroscopic analysis

Stationary absorption spectra of the purified proteins were recorded with a UV–Vis spectrophotometer (Shimadzu UV-2600, Japan), where the samples were kept at 0 °C using a cell holder equipped with a temperature-controlled circulating water bath. The CEL94649, VbACR2 and GtACR2 samples were suspended in Buffer B, Buffer B and Buffer E, respectively. We directly checked the absorbance values of the spectra and extracted the wavelength where the absorbance is the highest in visible light region (400–700 nm) as the λ_max_ of the rhodopsins.

Transient time-resolved absorption spectra of the purified proteins from 380 to 620 nm at 10 nm intervals were obtained using a homemade computer-controlled flash photolysis system equipped with an Nd:YAG laser as an actinic light source^30,56^. By using an optical parametric oscillator, the wavelength of the actinic pulse was tuned at 435 nm (4 ns) for VbACR2. The pulse intensity was adjusted to 2 mJ per pulse. The VbACR2 samples were kept at 25 °C using a cell holder equipped with a temperature-controlled circulating water bath and suspended in Buffer B. The time-dependent absorbance changes at 410 and 530 nm were fitted with a single-exponential function.

## Supplementary Information

Supplementary Information 1.

Supplementary Information 2.

## Data Availability

The source data underlying the main and supplementary figures and tables are shown as Supplementary data 1.
