# Supplementary material for: A blue-shifted anion channelrhodopsin from the Colpodellida alga Vitrella brassicaformis
Source: Sci Rep. 2023 Apr 28;13:6974. doi: 10.1038/s41598-023-34125-8 (PMC10147648; doi:10.1038/s41598-023-34125-8)
Supplement: Supplementary file 1 — Supplementary Information 1. [file 41598_2023_34125_MOESM1_ESM.docx]

**Supplementary Information**

**A blue-shifted anion channelrhodopsin from the** **Colpodellida alga *Vitrella brassicaformis***

Keiichi Kojima^1,2# *^, Shiho Kawanishi^2#^, Yosuke Nishimura^3^, Masumi Hasegawa^4^, Shin Nakao^2^, Yuya Nagata^2^, Susumu Yoshizawa^5^, Yuki Sudo^1,2 *^

^1^Faculty of Medicine, Dentistry and Pharmaceutical Sciences, Okayama University, Okayama 700-8530, Japan.

^2^Graduate School of Medicine, Dentistry and Pharmaceutical Sciences, Okayama University, Okayama 700-8530, Japan.

^3^Research Center for Bioscience and Nanoscience (CeBN), Research and Development Center for Marine Biosciences, Japan Agency for Marine-Earth Science and Technology (JAMSTEC), Kanagawa 237–0061, Japan

^4^Institute for Extra-cutting-edge Science and Technology Avant-garde Research (X-star), Japan Agency for Marine-Earth Science and Technology (JAMSTEC), Kanagawa 237–0061, Japan.

^5^Atmosphere and Ocean Research Institute, The University of Tokyo, Chiba 277-8564, Japan.

^#^These authors contributed equally to this work.

*To whom correspondence should be addressed

Keiichi Kojima; Telephone: +81-86-251-7980, E-mail: keiichikojima@okayama-u.ac.jp

Yuki Sudo; Telephone & FAX: +81-86-251-7945, E-mail: sudo@okayama-u.ac.jp


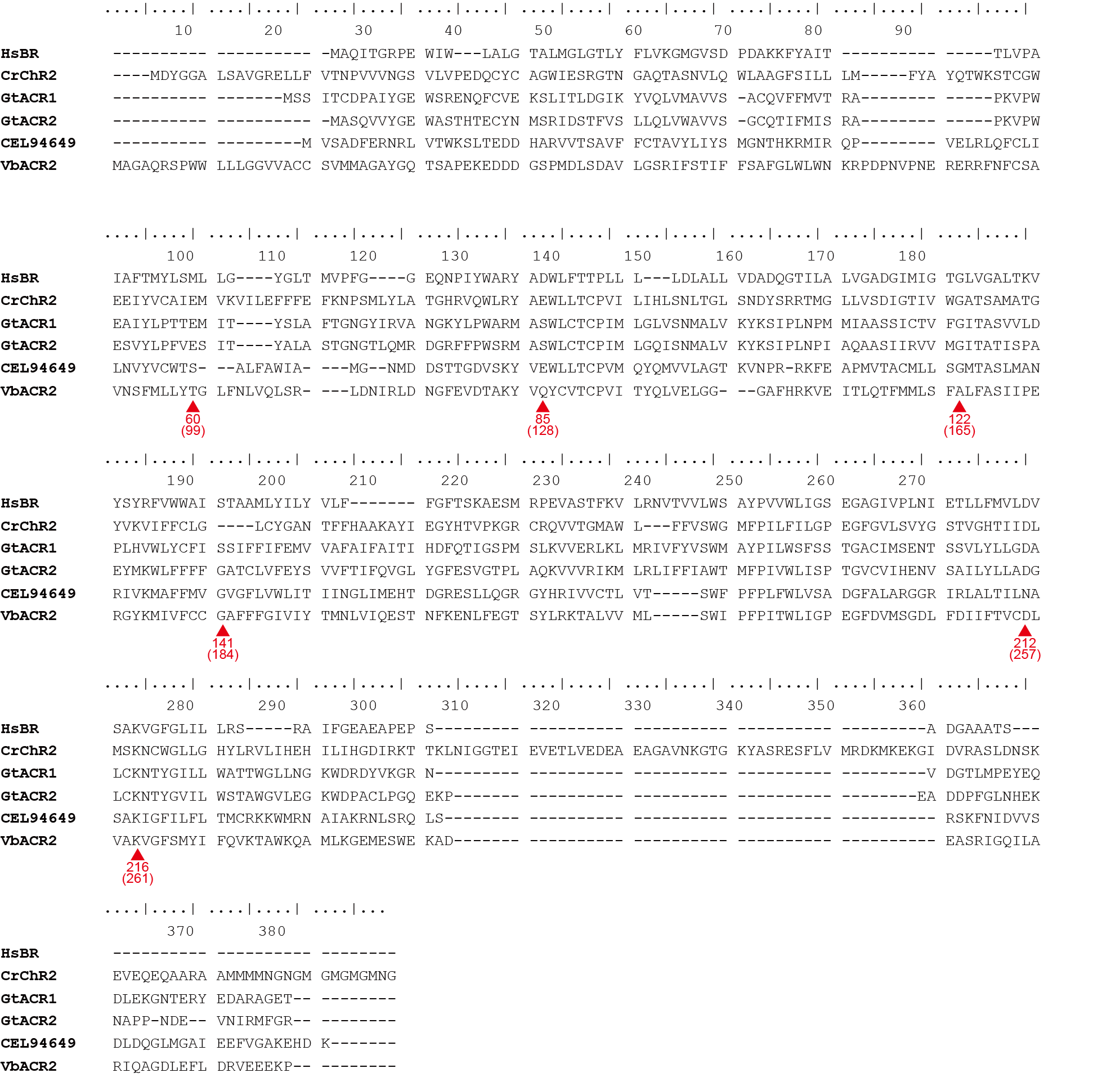


**Figure S1**. **Amino acid sequence alignment of CEL94649, VbACR2 and known microbial rhodopsins**

Accession numbers of the sequence data are as follows: HsBR, CAP14056; CrChR2, AAM15777; GtACR1, KP171708; GtACR2, KP171709; CEL94649; and VbACR2, CEM28120. The numbers of amino acids in HsBR and in VbACR2 (in parentheses) are indicated below the columns (red characters).


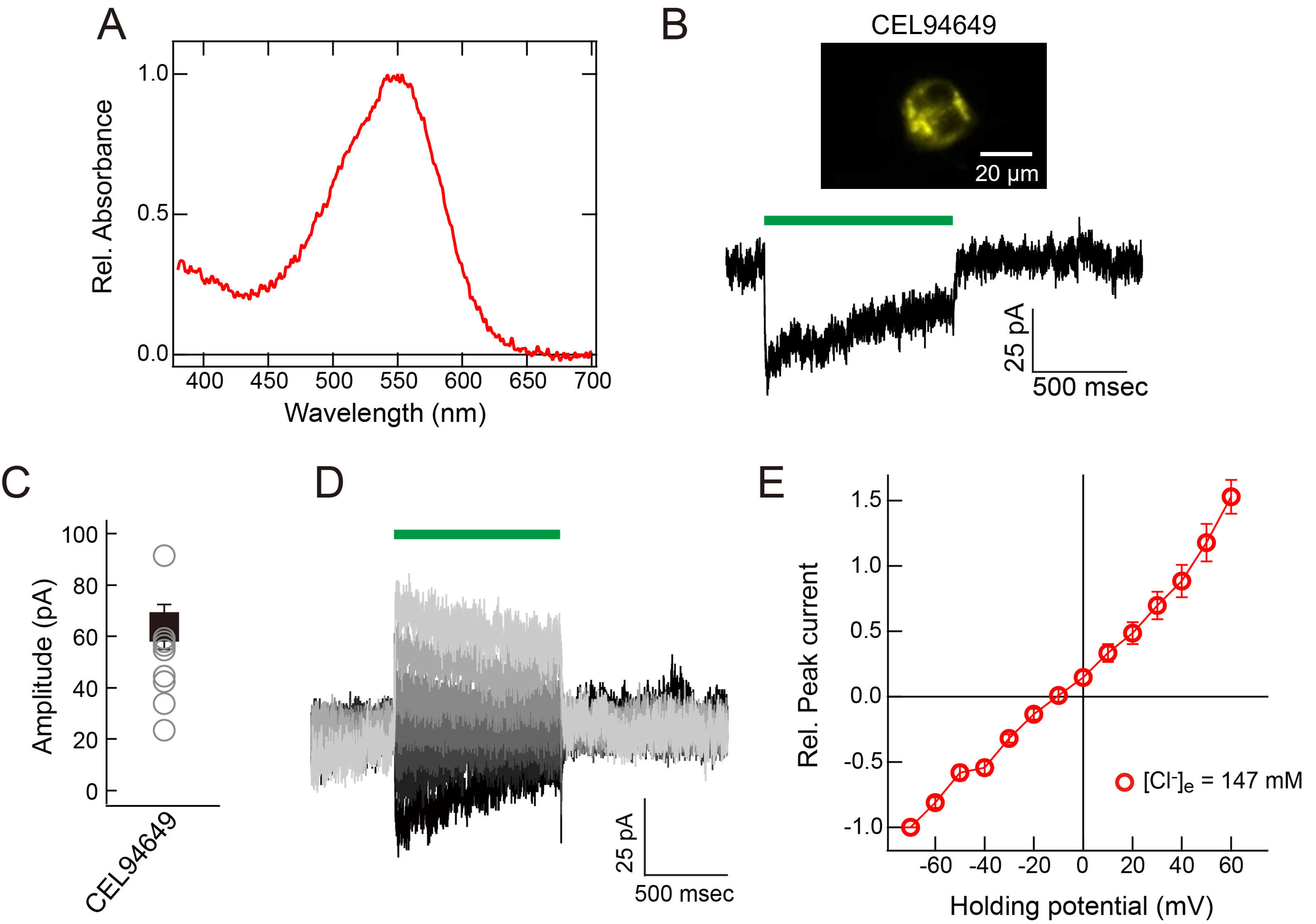


**Figure S2**. **Spectroscopic and electrophysiological measurements of CEL94649**

(A) The absorption spectrum of the purified CEL94649 in SMA lipid particles. The spectrum was normalized at peak absorbance. (B) Representative photocurrent trace of CEL94649 elicited with green light (green bar) at the membrane potential of -60 mV in the standard intracellular and extracellular solutions (146 and 147 mM Cl^-^ in the intracellular and extracellular solution, respectively) (Table S1). Fluorescence image of ND7/23 cell expressing CEL94649 was also shown. The fluorescence signals derived from EYFP which was fused to the C-terminus of CEL94649 were monitored. (C) Peak current amplitudes of CEL94649 elicited with green light at the membrane potential of -60 mV in the standard intracellular and extracellular solutions. The data are mean values ± SEM (n = 11 cells). (D) Representative photocurrent traces of CEL94649 elicited with green light (green bar) at different membrane potentials (-60 to +60 mV, in 20 mV steps, from the bottom to the top) in the standard intracellular and extracellular solutions. (E) Current-voltage relationship of the CEL94649 peak photocurrent in the standard intracellular and extracellular solutions. Peak currents were normalized at the membrane potential of -70 mV. The data are mean values ± SEM (n = 10 cells).


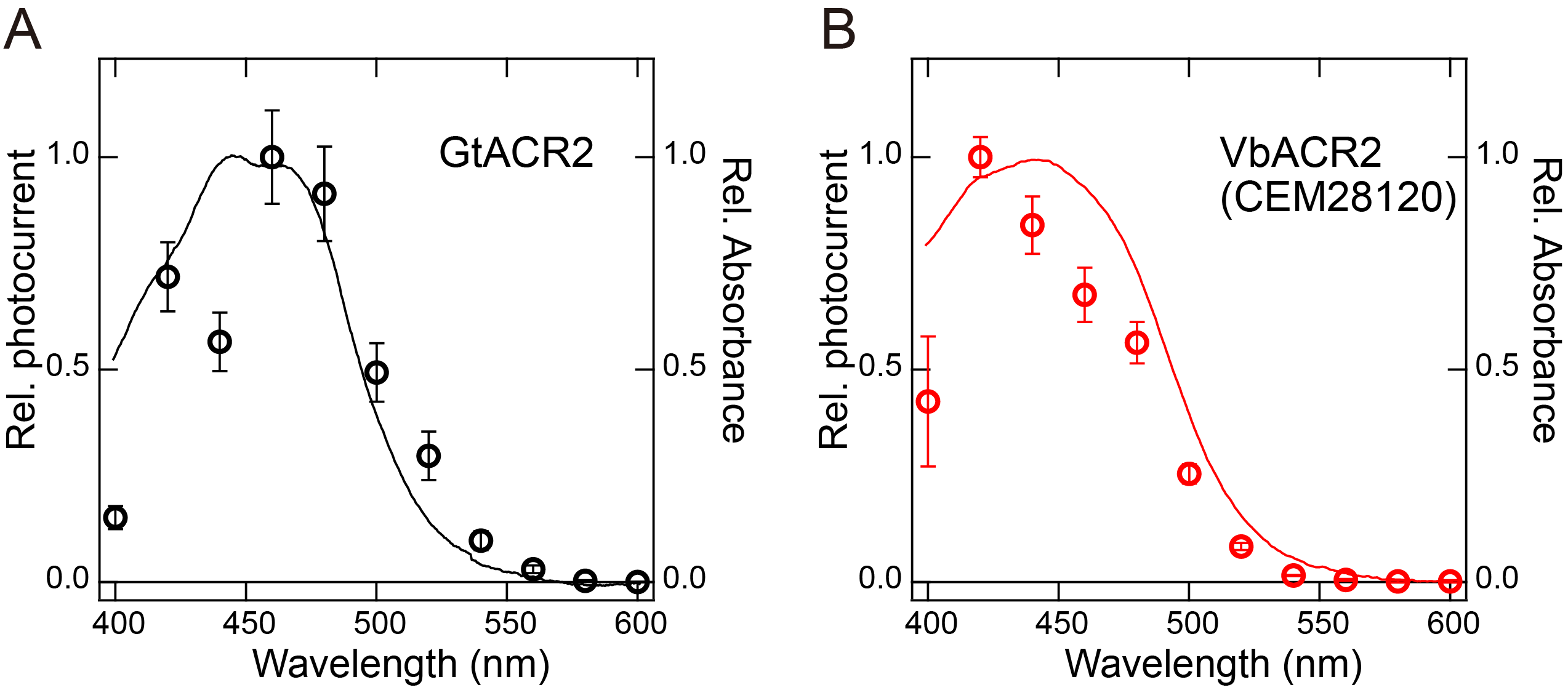


**Figure S3**. **Comparison of photocurrent action spectra and absorption spectra of GtACR2 and VbACR2**

The peak current amplitudes of GtACR2 (A) and VbACR2 (B) were plotted against the wavelengths of light. The absorption spectra of GtACR2 (A) and VbACR2 (B) were shown as black and red lines, respectively, for comparison.


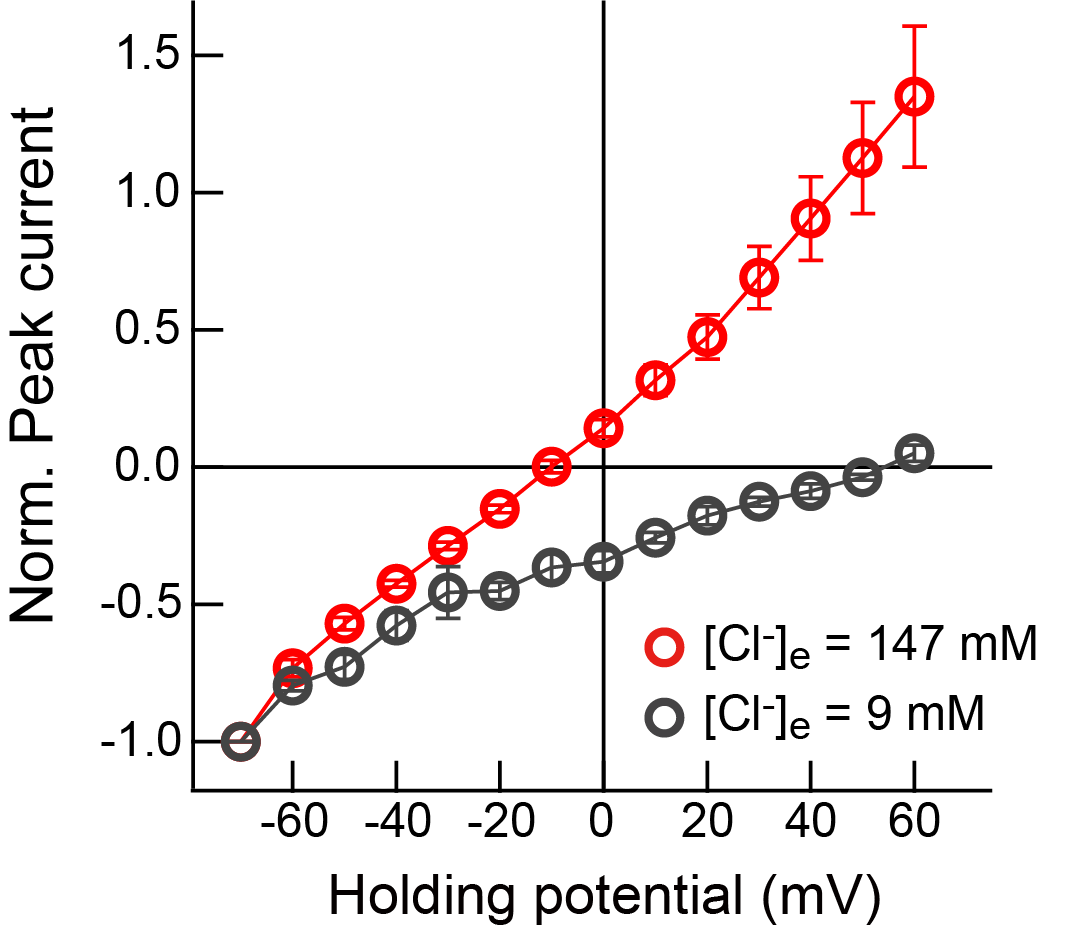


**Figure S4**. **Current-voltage relationship of VbACR2 with the intracellular solution containing 150 mM Na^+^**

Current-voltage relationship of the VbACR2 (CEM28120) peak photocurrent upon replacing NaCl with sodium gluconate in the extracellular solution. The photocurrents were measured with the Na^+^ intracellular solution (Table S1). Peak currents were normalized at the membrane potential of -70 mV. The data are mean values ± SEM (n = 5-6 cells).


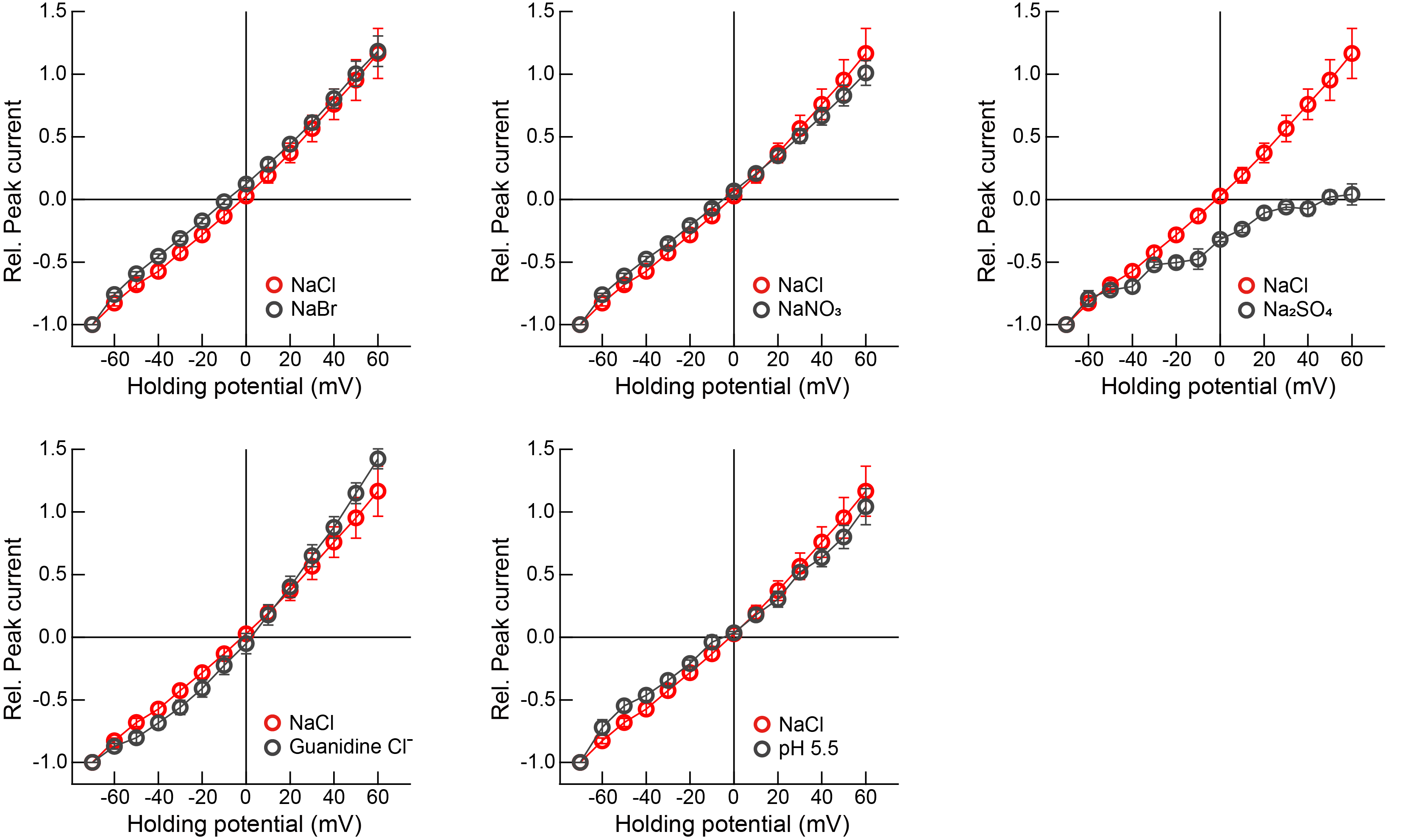


**Figure S5.** **Current-voltage relationship of VbACR2 in different extracellular solution conditions**

Current-voltage relationship of the VbACR2 (CEM28120) peak photocurrent upon exchange of the extracellular solution. The photocurrents were measured with the standard intracellular solution (Table S1). Peak currents were normalized at the membrane potential of -70 mV. The data are mean values ± SEM (n = 5-7 cells).

**Table S1 Composition of intracellular solutions in experiments with ND7/23 cells.**

All concentrations are in mM.

|  | Standard solution | Na^+^ solution |
| --- | --- | --- |
| HEPES | 50 | 50 |
| CsCl | 140 | - |
| NaCl | - | 140 |
| MgCl_2_ | 3 | 3 |
| Na_2_EGTA | 5 | 5 |
| MgATP | 2.5 | 2.5 |
| pH | 7.3 | 7.3 |

**Table S2 Composition of extracellular solutions in experiments with ND7/23 cells.**

All concentrations are in mM. Liquid junction potentials (LJPs) are listed in mV.

|  | Standard solution | Gluconate solution | Br solution | NO_3_ solution | SO_4_ solution | Guanidine Cl^-^ solution | Acidic solution |
| --- | --- | --- | --- | --- | --- | --- | --- |
| HEPES | 10 | 10 | 10 | 10 | 10 | 10 | 10 |
| NaCl | 138 | - | - | - | - | - | 138 |
| Na gluconate | - | 138 | - | - | - | - | - |
| NaBr | - | - | 138 | - | - | - | - |
| NaNO_3_ | - | - | - | 138 | - | - | - |
| Na_2_SO_4_ | - | - | - | - | 92 | - | - |
| Guanidine Cl^-^ | - | - | - | - | - | 138 | - |
| KCl | 3 | 3 | 3 | 3 | 3 | 3 | 3 |
| MgCl_2_ | 1 | 1 | 1 | 1 | 1 | 1 | 1 |
| CaCl_2_ | 2 | 2 | 2 | 2 | 2 | 2 | 2 |
| glucose | 100 | 100 | 100 | 100 | 100 | 100 | 100 |
| pH | 7.3 | 7.3 | 7.3 | 7.3 | 7.3 | 7.3 | 5.5 |
| LJP with Standard solution | 8.3 | 0.1 | 8.7 | 7.3 | -0.2 | -6.0 | 8.3 |
| LJP with Na^+^ solution | 3.6 | -11.2 | - | - | - | - | - |
